# Spavin: Its Etiology and Treatment1Read before the American Veterinary Medical Association, September 5, 1900.

**Published:** 1901-02

**Authors:** W. J. Martin

**Affiliations:** Kankakee, Ill.


### SPAVIN: ITS ETIOLOGY AND TREATMENT.[1]

BY W. J. MARTIN, M.D.C.,
KANKAKEE, ILL.

*Etymology of the word spavin.* Old French, *esparvain;* new French, *esparvin;* Spanish, *esparavan;* Italian, *spavenio* and *spavento.* From old French, *espervien;* old high German, *sparwari,* a sparrow hawk, so named from the peculiar gait of a horse lame from this disease, which makes the animal raise the lame leg like a sparrow hawk. (*Webster's Dictionary* and the *Standard Dictionary.*)

*Definition.* Spavin is an inflammatory ostitis affecting the component structure of the bones and also of the ligamentous tissues

[1] Read before the American Veterinary Medical Association, September 5, 1900.

that compose the equine tarsus, and which sooner or later (in the majority of cases, at least) produces ossific exostosis and ankylosis of two or more bones of the articulation. This ankylosis may be partial or complete. The ostitis and accompanying exostosis usually first appear at the inner angle of the cuneiform magnum and the articular facets of the parvum and the inferior surface of the scaphoid bones, though quite frequently the disease involves the articulating surface of the scaphoid, inferior surface of the astragalus and cuboid bones. Another, though less common, seat of the disease is the surfaces of articulation between the astragalus and os calcis, and in this location the disease usually partakes of the nature of a true form of osteoarthritis, and often terminates in caries and necrosive ulceration within the joint.

*Pathology.* The inflammation occurring in spavin may be considered of two distinct forms, namely, acute and chronic. The disease may arise from two causes, namely, idiopathic and traumatic. Under the first head may be included all hereditary predispositions to osseous exostosis, due primarily to defective anatomical construction of the skeletal framework, the usual result of which is endositis, periostitis, and osseous exostosis of the bones of the articulation. Under the second head we include all external agencies that directly or indirectly cause the disease, such as concussion, severe strain or stretching of the ligaments of the joint, more especially the interosseous ones; injuries, such as kicks, wounds, etc., though in my experience spavin is rarely due to injuries of a direct nature, though an injury may be the indirect cause of spavin, as where the opposite limb has been injured and the animal in favoring it throws the entire weight of the posterior part of the body upon the sound limb, which in time may become affected with spavin.

The question is often asked, Why does spavin most generally appear at the junction of the cuneiform magnum and parvum? In viewing a tarsus in profile we notice that the bones of the joint do not rest upon a plane surface, but incline gradually from the external part of the joint to its internal portion. We also note a greater development of the bones composing the external part of the tarsus than those of the internal portion. This removes slightly the line of gravity from the central part of the joint toward its inner part, and as a result the line of weight descending from the tibia to the surface of the astragalus is thrown in an oblique manner mostly upon the inner portion of that bone, and is in turn transmitted to the inner part of the scaphoid, cuneiform magnum, and parvum. The parvum being the slightest constructed bone in the joint, naturally is the first to become the seat of inflammation from the unequal distribution of weight.

The divergent views held by some of the most eminent members of the profession in regard to the pathology of spavin renders the subject a most difficult one for the general practitioner to approach or even to venture an opinion on. Some hold that it is the ligaments of the joint in every case that are first to become the seat of the inflammatory action, and from them it next extends to the substance of the bones. Others claim that the disease invariably originates in a strain of the inner tendon flexor metatarsi muscle; while others claim, among them being the distinguished Dieckerhoff, that the inflammation is first set up in the sheath of the tendon of the flexor metatarsi, though why inflammation of this sheath should cause spavin, when it rarely causes even slight lameness, appears strange to me. Our own opinion is that spavin is nothing more or less than an ostitis of the bones of the hock-joint. The ostitis may originate within the cancellated structure of the bone (endositis) or on the external surface of the bone (periostitis). Endositis is the form present in idiopathic spavin, while the periostitic form is due to traumatic causes, such as strains of the ligamentous structure of the joint, from concussion, etc.

In endositic inflammation of the bones of the tarsus the delicate membrane lining the Haversian canals becomes highly congested and inflamed. This inflammation in time spreads from within outward and affects the space between the compact bone and the periosteum. As the articular cartilage of a joint may, for all practical purposes, be considered a continuation of the periosteum, it can be readily seen how endositis may rapidly spread throughout an articulation. One important fact in connection with the pathology of spavin or other forms of bone disease should be borne in mind, and that is "that it is in the soft parts of bone, the periosteum, the medullary membranes, and the delicate connective tissues which pervade the Haversian canals and cancelli that the inflammation occurs, and that the pathological process, though somewhat modified by the hard and resisting nature of the osseous framework, is essentially similar to that which occurs in the soft tissues."

In the endositic form of spavin as the inflammation advances the peripheral lamellæ and articular cartilage become involved in the destructive metamorphosis, in which the canaliculi of the opposing bones are laid bare by the constant friction and irritation, thus establishing a vascular communication between the Haversian canals and lacunæ of the bones. These vascular openings pour out an effusion of bone-forming lymph, which in time firmly cements the diseased bones together. We see this condition finely illustrated in the specimen before you, in which it will be seen that the scaphoid, cuneiform magnum, and parvum are firmly cemented together without there being the slightest periostitic enlargement on the external borders of the bones.

In the periostitic form of spavin the inflammation advances from the periphery of the bones to their centre, and, as before mentioned, is usually due to external influences, such as strain of the interosseous ligaments, articular arthritis, violent overcontraction of muscles or tendons implanted in the periosteum of the bones. "The pathological changes in circumscribed periostitis consist of an inflammatory transudation into both the superficial and the deeper strata of the membrane, forming an enlargement sometimes termed a 'node.' This transudation, consisting of fibrous lymph and leucocytes, serves to form a prominence over the surface of the bone." (Agnew.) This inflammatory node in the horse when affecting the bones of the tarsus constitutes the osseous exostosis termed spavin.

*Etiology.* Spavin, a disease due to domestication. Spavin is rarely seen among equidæ living in a state of nature. The horses that live on the plains and in the valleys of the Rocky Mountain regions of the United States, Canada, and Mexico are rarely seen to be affected with spavin. I am reliably informed that among horses running wild on the pampas of South America it is a very rare sight to see one affected with spavin.

During the several years in which I have been engaged in the study of the osteology of fossil equidæ I have examined the hock bones of fossil horses from nearly all parts of the world without being able to discover any trace of a disease among them similar to spavin in the domestic horse. It is true that these fossil horses are of a distinct genera to that of the domestic species, yet the bones of the hock are almost identical with those of the domestic animal. More especially is this true of Hipparion.

From a close study of the pathology of spavin it will be seen that it often becomes a difficult matter to ascertain the true cause of spavin. In the idiopathic form heredity undoubtedly plays an important part, because there is probably no domestic animal so prone to morbid ossific enlargement as the horse.

In my opinion the most common cause of spavin is a defective anatomical conformation of the bones of the entire limb as well as those of the tarsus, and in tracing the cause of spavin lameness we should view the limb as a whole rather than at any one point. We should note the formation of the muscles of the limb, the manner in which they perform their functions, as well as their relation to the bones of the limb; in a word, take a generalized view of the whole mechanical construction of the diseased leg.

In examining a hock affected with spavin we notice in the majority of cases a peculiar rounded form of the superior head of the large metatarsal bone. This is anomalous, and gives to the scaphoid and cuneiform bones the appearance of being top-heavy; we almost invariably find such joints the seat of spavin. In the bent or "sickle hock" the powerful action of the extensor muscles of the limb when the animal is subjected to hard and fast work will, by the great pressure and contraction on the articulating surfaces of the bones and ligaments, excite ostitic inflammation. Some pathologists claim that spavin is due to incomplete formation of the bones of the hock, and, hence, should be viewed as simply a neoformation of bone to complete the bone-forming process.

It is claimed by some German authors that large horses with powerfully developed muscles of the hind-quarters are, from the powerful mechanical action of flexion and extension of the limb on the hock, much predisposed to spavin; also, that long, weak-backed animals are often affected with the disease. I have often seen spavin occur in mares that had passed through a severe attack of parturient laminitis, in which the major portion of the weight of the body was thrown upon the posterior part of the body. In this affection articular arthritis is a common sequel and often ends in ostitis and ankylosis.

*Semiology.* Mr. Percivall, in speaking of the symptoms of spavin, says: "The symptoms of spavin are, in general, plain and simple and unequivocal." With all due deference to such a distinguished author, I must say that in my experience, where the exostosis is not yet present, spavin is one of the most difficult forms of lameness to properly diagnose. There is, perhaps, no other form of lameness of the horse in which the practitioner should be more guarded in his expression of an opinion—moreover, the young and inexperienced one.

The idea that certain characteristic movements of the hock-joint are always indicative of spavin lameness is, in a great many cases, erroneous. A much more certain sign of spavin lameness would, in my opinion, be the wearing away of the toe of the shoe or hoof, as this condition is almost invariably present in cases of spavin lameness. Where the exostosis is not present and the ostitis is not extensive, being confined to the inner angle of the scaphoid and the cuneiform bones, the diagnosis can be made certain only by inference, such as the history of a slight lameness which would disappear after a certain amount of exercise had been taken, the absence of any pathological lesion around the foot, such as ring-bone, side-bones, tendonitis, curb, patellar lameness (gonitis chronica), hip-joint disease, or long-standing lameness with atrophy of the gluteal and patellar muscles, from repeated attacks of azoturia, etc. All these conditions should be taken into consideration by the practitioner.

The so-called " spavin test," in which the limb is strongly flexed and immediately after the animal is made to trot briskly, is, in my opinion, nearly worthless for diagnostic purposes. I have seen this test applied to horses that were perfectly sound, and yet they would move off quite stiffly for a short distance, and markedly so if aged. Atrophied muscles in the patellar and gluteal regions are rarely due to true bone spavin, this condition being most often seen in cases affected with rheumatoid arthritis of the hock and with gonitis chronica of the patella.

A horse suspected of spavin lameness should if possible be seen by the practitioner while at rest in the stable. It will there be observed that the animal will flex the suspected limb much more than its fellow. This is done in a peculiar manner. The point of the toe is carried outward, and the heel of the foot, owing to the action of the abductor and adductor muscles of the limb, will be rested upon the upper part of the opposite hoof. At this time, if the animal is made to move quickly over in the stall on the sound limb, the movement will be accomplished with a sort of hopping action, and the lameness will be quite marked, as a rule. The animal moves with much more facility when made to move toward the lame leg.

When the animal is trotted to halter the lameness is marked by a spasmodic or convulsive contraction and flexion of the hock-joint and a rising and falling of the hip on the lame side. The limb when first placed upon the ground will rest upon the toe, but as the exercise is increased the animal will gradually place more and more of the surface of the foot upon the ground until he is able to place the entire weight upon the heels. During this movement the leg, when carried in extension, does not move through the full segment of its circle of extension and flexion as in a healthy joint. This conditional movement is most marked just prior to the appearance of the exostosis.

*Ocular Inspection and Palpation.* In order to better observe the presence of an exostosis upon a suspected joint, have the animal placed upon a level footing and with both hind legs as nearly together as possible; stand several yards in front of the animal, and, looking between the front legs, compare the outline of each joint with that of the opposite one; carefully note any irregularities of the lame joint and compare them closely with the bones of the sound one. Then step to one side, nearly on a line with the shoulder of the sound side and *vice versa*, when any enlargement on the surface of the lame leg can be readily detected. Palpation for spavin between the scaphoid and cuneiform bones of the joint, and when the exostosis is not well marked, is of but very little value. When the ostitis is confined to the articulating facets of the astragalus and os calcis, palpation by the trained and sensitive finger of the surgeon will readily detect any existing inflammation in the parts.

The question is often asked, How long a period of time will elapse from the beginning of spavin lameness until the exostosis will become visible? In some rare instances but a few weeks will pass before the exostosis is well developed, though in my experience this is an exception and not the rule. In most of the cases coming under my observation the lameness may exist for several months before the exostosis becomes perceptible, and I recall one case in particular in which there was persistent lameness for three years before the exostosis made its appearance.

In pronouncing a horse spavined in which there is no visible exostosis present we are often met by the assertion of interested parties that if the horse has a spavin coming on the lame leg he must certainly have one on the well leg, as both joints are exactly alike. I wish to state, more especially for the benefit of the young practitioner, that in horses with coarse hocks and in which there is an undue prominence of the borders of the scaphoid and cuneiform bones, and even if there is not the slightest lameness, he can, in nearly every case, safely make the statement that such a hock is already affected with spavin. It is rare in my experience to find an animal affected with spavins on both hocks to be lame in more than one leg at a time. Ankylosis of the bones of the hock without lameness is by no means confined to adult animals. I have seen well-marked exostosis and ankylosis of the scaphoid and cuneiform bones in a colt but a few days old. This diseased condition can be accounted for in no other manner than that the diseased state must have taken place during the intra-uterine period of its existence.

*Treatment.* The treatment of spavin has varied but little from the fundamental methods laid down by Youatt and Percivall, and that this method was based upon a correct scientific knowledge of the pathology of the disease is borne out by the experience of nearly every practitioner from their time to the present. The most essential requirement in the treatment of spavin as well as in all other joint diseases is rest. By rest I do not mean the turning of the animal out in pasture, where, by running and jumping in play, the animal has every facility for keeping the diseased bones in a state of constant irritation. If a small box-stall with a dirt floor is not available I have the animal placed in an ordinary single stall. Bring the feet into proper shape by levelling and proper shoeing. There is no particular pattern of shoe required for this purpose beyond that which will place the bones of the joint upon an even surface.

In the early stage of the disease if there is severe inflammatory action present, applications of hot or cold water may be made to the joint, though usually I consider this a waste of valuable time, because the majority of the cases met with have already passed through the acute stage. It is an incontrovertible fact that horses in country districts are more easily treated and cured of spavin than are horses in cities ; also, that the exostosis is much smaller in the former than in the latter.

The question is often asked, Should we make any distinction in the choice of a therapeutic agent when treating the ostitis or periostitis existing in a hock-joint? I should answer, no. While articular arthritis existing between the astragalus and os calcis is a much more severe disease than where the disease is confined to the border of the cuneiform bones, yet the therapeutic agent that will relieve the one will answer for the other equally well.

What forms of therapeutic agents are the most efficacious in combating spavin? We answer, the chemical ones, such as mercury, iodine, the mineral acids, etc. Cantharides, which occupies a prominent place in many of our text-books for the treatment of spavin, is, in my opinion, of very little value for this purpose.

Our object in applying chemical agents to a joint affected with spavin is, as every practitioner knows, not with the object of removing the osseous exostosis, but merely to assist nature in hastening the process of ankylosis, because a joint once the seat of inflammatory ostitis can never be restored to its pristine condition.

Of the above-mentioned chemicals I prefer the bichloride of mercury in solution, supplemented by camphor and oil of turpentine. The following are the formulæ: ℞.—Hydrarg. chlor. corrosive, one ounce; sp. vini. rect., seven ounces; hydrochloric acid, one ounce.—M. Add the acid to the spirit and dissolve the mercury in the mixture, then add: sp. vini. rect., thirty-six ounces; gummi camphoræ, four ounces; olei terebinthinæ, six ounces.—M. This fluid is dispensed in six or eight-ounce bottles with instructions to apply a portion to the joint once or twice a day for a period of from two to four weeks if necessary. I have found that this compound has a tendency to relieve quickly the pain of spavin; and, furthermore, it is a much more satisfactory method than that of firing every case in the beginning of the disease, as many cases will recover without blemish under this form of treatment. I find in country districts a considerable prejudice against the use of the hot iron in treating spavin, and in the case of valuable animals the owners will often resort to nearly every other means before resorting to its use. However, I am a firm believer in the efficacy of the hot iron in the treatment of spavin after other remedial agents have failed. The pointed pear-shaped iron is the one preferred by me, and during the operation it is introduced deeply into the exostosis.

*Cunean Tenotomy.* In cases in which the exostosis is very large this operation often proves of much benefit by lessening the intense pain and irritation of the mechanical pressure of the fibres of the metatarsi muscle over the osseous enlargement. The operation is quite simple and requires but very little attention afterward.

*Tibial Neurectomy.* Resection of the tibial nerve for the cure of spavin has been extensively practised by European veterinarians with somewhat varying degrees of success, but at the present time the operation is not thought to be of much benefit. Prof. Bosi, of the Bologna Veterinary School, has lately recommended a double neurectomy at the hock for the cure of spavin, and it is claimed that 90 per cent. of the cases so operated on have been cured. The operation consists of a resection of the tibial nerve and also of the peroneal nerve.[1]

[1] See American Veterinary Review for August, 1900.

